# Context-dependent efficacy of fibroblast activation protein-targeted radionuclide therapy (FAP-TRT): the dual role of CAFs

**DOI:** 10.1007/s00259-026-08099-4

**Published:** 2026-07-29

**Authors:** Chunhui Wu, Ahmad Kurniawan, Ge Li, Zihe Ming, Maarten W. Nijkamp, Evert van den Broek, Walter Noordzij, Andor W. J. M. Glaudemans, Ines F. Antunes, Bart Cornelissen

**Affiliations:** 1 Department of Nuclear Medicine and Molecular Imaging, University of Groningen, University Medical Center Groningen, Groningen, the Netherlands; 2 Department of Surgery, University of Groningen, University Medical Center Groningen, Groningen, the Netherlands; 3 Department of Pathology and Medical Biology, University of Groningen, University Medical Center Groningen, Groningen, the Netherlands

**Keywords:** Fibroblast activation protein, Targeted radionuclide therapy, Cancer-associated fibroblasts, Pancreatic ductal adenocarcinoma, Tumour microenvironment

## Abstract

**Abstract:**

Fibroblast activation protein-targeted radionuclide therapy (FAP-TRT) shows promise across multiple cancers, but clinical responses remain variable. Here, we aim to investigate how the presence, abundance, and spatial organisation of FAP-expressing cancer-associated fibroblasts (CAFs) relative to tumour cells influence the efficacy of FAP-TRT, to further optimisation.

**Methods:**

Spatial heterogeneity of FAP expression and radioligand uptake was assessed in human pancreatic ductal adenocarcinoma (PDAC) tissues using immunofluorescence and autoradiography with [^1^⁶^1^ Tb]Tb-FAPI-46. Mechanistic studies were conducted in vitro using PSN-1 tumour cells and FAP-expressing CAFs in 2D cultures and 3D spheroids, including mono- and co-culture systems with defined tumour-to-CAF ratios and spatial configurations. Cellular uptake and nuclear absorbed dose were quantified, and radiobiological effects were evaluated using DNA damage (γH2AX), clonogenic survival, spheroid growth, and cytokine profiling.

**Results:**

PDAC tissues exhibited pronounced spatial heterogeneity in FAP expression and radioligand uptake. In vitro, uptake alone did not predict absorbed dose or biological response. In 2D models, [^1^⁶^1^ Tb]Tb-FAPI-46 did not significantly reduce clonogenic survival or increase DNA damage response. In 3D spheroids, FAP-TRT induced dose-dependent DNA damage and growth inhibition. Mixed tumour–CAF spheroids showed more uniform DNA damage and stronger growth suppression than layered models. CAF-containing models attenuated tumour-cell DNA damage and growth inhibition compared to models without CAFs (*P* < 0.001), associated with increased IL-6 and TGF-β secretion.

**Conclusion:**

FAP-expressing CAFs have a dual, context-dependent role in FAP-TRT, enhancing tumour irradiation through crossfire while also limiting tumour control potentially through radioligand partitioning towards the stromal compartment and CAF-derived paracrine signalling. These findings identify spatial organisation and CAF radiobiology as key determinants of FAP-TRT efficacy.

**Supplementary Information:**

The online version contains supplementary material available at https://doi.org/10.1007/s00259-026-08099-4.

## Introduction

Targeted radionuclide therapy (TRT) has emerged as a promising strategy for patients with unresectable, recurrent, or metastatic cancer [1–3]. TRT relies on the selective delivery of radiopharmaceuticals to tumour-associated molecular targets, enabling localised deposition of ionising radiation within tumour lesions [3]. Fibroblast activation protein (FAP) is overexpressed on cancer-associated fibroblasts (CAFs) in the stroma of a wide array of tumours and is therefore an attractive pan-tumour target [4–6]. In multiple tumour types, FAP-positive CAFs are associated with aggressiveness and resistance to chemotherapy and radiotherapy [7, 8], supporting the concept that FAP-directed approaches could simultaneously provide diagnostic information and enable therapeutic intervention. Accordingly, FAP-targeted radioligands have rapidly advanced from imaging to therapeutic applications, with FAP-TRT expected to act through irradiation of FAP-expressing stromal elements and crossfire irradiation of adjacent tumour cells [9, 10].

Despite encouraging preclinical activity across diverse animal models [11–13], the clinical efficacy of FAP-TRT has so far been heterogeneous, with most studies reporting relatively low objective response rates (ORRs). Median ORR across all published FAP-TRT studies is approx. 20%, with most studies reporting ORRs well below 30% and several showing no partial responses at all despite significant uptake [14–22]. The factors shaping the efficacy of FAP-TRT can be broadly grouped into three sets: (i) the radiopharmaceutical itself, including radionuclide selection and ligand design; (ii) tumour heterogeneity; and (iii) radiobiological interactions between the radiopharmaceutical and the tumour microenvironment (TME). To date, most FAP-TRT studies have primarily focused on component (i), for example, by optimising ligand structures to improve tumour retention [12, 23–26] or by pairing FAPI derivatives with radionuclides of different emission properties and linear energy transfer (LET) to enhance tumour absorbed dose [13, 27, 28]. Previous cellular dosimetry studies have shown that ^1^⁶^1^ Tb-labelled peptides can deliver higher nuclear absorbed doses than their ^1^⁷⁷Lu-labelled counterparts at comparable uptake [29], mainly due to additional internal conversion electron emission, supporting its use for investigating cellular and subcellular determinants of FAP-TRT efficacy. In contrast, the contributions of intratumoural heterogeneity and TME-driven radiobiology to therapeutic response remain comparatively underexplored. Among FAP-expressing solid tumours, pancreatic ductal adenocarcinoma (PDAC) provides a particularly relevant model for studying FAP-TRT because it is a prototypical desmoplastic malignancy characterised by abundant CAF-rich stroma, frequent FAP expression, pronounced tumour–stroma heterogeneity, and a major unmet therapeutic need [30, 31].

Accordingly, this study aims to identify key determinants of FAP-TRT efficacy, with an emphasis on how heterogeneity in FAP expression and radioligand uptake may translate into spatially uneven radiation dose deposition, and how FAP-expressing CAFs modulate radiobiological responses beyond uptake alone. To address these questions, we combine spatial profiling of PDAC patient tissues with mechanistic *in vitro* 2D and 3D models treated with [^161^ Tb]Tb-FAPI-46.

## Materials and methods

### Ethical approval and sample selection

For the current study, ethical approval was obtained from the Central Ethics Review Board non-WMO studies from the UMCG (Centrale Toetsingscommissie UMCG) under research number 2022000293. Informed consent was waived for this study. Human PDAC samples were obtained from ten consecutive chemotherapy-naïve patients undergoing pancreatic resection for PDAC in the UMCG between February 2022 and December 2022. The diagnosis of PDAC was histologically confirmed by an experienced pathologist. The study was conducted in accordance with the Declaration of Helsinki.

### Immunofluorescence staining and autoradiography of pancreatic ductal adenocarcinoma (PDAC) sections

Immunofluorescence staining for FAP and α-SMA was performed on formalin-fixed paraffin-embedded (FFPE) PDAC sections (*n* = 10) to assess spatial patterns of FAP expression in stromal and tumour compartments. Antigen retrieval, antibody incubation, and confocal imaging were performed using standardised protocols. Detailed staining procedures are provided in the Supplementary Information (SM. 1).

### Cell culture and CAFs activation

PSN-1 human PDAC cells and VH-25 primary human fibroblasts were used in this study. PSN-1 cells were cultured in RPMI 1640 supplemented with foetal bovine serum and standard additives, whereas VH-25 fibroblasts were maintained in DMEM-based medium. CAF-like activation of fibroblasts was induced by exposure to PSN-1–conditioned medium for 4 days, resulting in elevated FAP expression. Cell identity and culture quality were routinely verified. Detailed procedures are provided in the Supplementary Information (SM. 2).

### Radioligand synthesis

[^1^⁶^1^ Tb]Tb-FAPI-46 was synthesised with high radiochemical purity as described in the Supplementary Information (SM. 3).

### *In vitro* uptake, internalisation, and specificity (blocking), efflux (cellular retention)

Cellular uptake, internalisation, efflux, and specificity of [^1^⁶^1^ Tb]Tb-FAPI-46 were assessed in PSN-1 and CAFs under low and high activity conditions, as described in the Supplementary Information (SM. 4).

### Single-cell dosimetry and nuclear absorbed dose estimation

Radiation absorbed dose to the nucleus was estimated using the MIRDcell software package (V4.16), as described in Chan et al. [32].

### Clonogenic survival (colony formation) assay

To assess radiotoxicity in 2D monolayer cultures, clonogenic survival assays were performed using PSN-1 monocultures and PSN-1/CAF co-cultures at tumour-to-CAF ratios of 1:2 and 1:5. Cells were exposed to increasing activities of [^1^⁶^1^ Tb]Tb-FAPI-46, replated at low density, and cultured to allow colony formation. Colony numbers were quantified as a measure of long-term clonogenic survival. Detailed procedures are provided in the Supplementary Information (SM. 5).

### 3D spheroid models and radionuclide treatment

To model tumour–stroma spatial organisation, 3D spheroids were generated using PSN-1 cells alone or co-cultured with CAFs at defined ratios (1:2 or 1:5). Co-culture spheroids were established using either layered or mixed configurations to reflect different spatial arrangements of tumour cells and CAFs. After spheroid formation, cultures were treated with [^1^⁶^1^ Tb]Tb-FAPI-46 at increasing activity levels, followed by long-term monitoring of spheroid growth. Spheroid size was quantified over time and normalised to baseline measurements. Detailed procedures are provided in the Supplementary Information (SM. 6).

### DNA damage assessment by immunofluorescence (IF)

Radiation-induced DNA damage was evaluated by IF detection of γH2AX in both 2D cultures and 3D spheroids. For spheroids, γH2AX distribution was analysed in central optical sections to assess spatial patterns of DNA damage in relation to CAF abundance and organisation. Detailed staining and image analysis workflows are provided in the Supplementary Information (SM. 7).

### Conditioned-medium (CM) and cytokine analysis

To investigate CAF-mediated paracrine effects during FAP-TRT, conditioned-medium experiments were performed using supernatants collected from irradiated CAF spheroids. The effects of conditioned media on PSN-1 spheroid growth were subsequently evaluated. Cytokine levels (including IL-6, TGF-β, and VEGF) were quantified from spheroid culture supernatants on day 13 using multiplex immunoassays. Detailed procedures are described in the Supplementary Information (SM. 8).

### Statistical analysis

All statistical analyses were performed using GraphPad Prism 10 (version 10.1.1 or higher). Individual data points are shown for all experiments. Data normality was assessed using the Shapiro–Wilk test. For datasets that were approximately normally distributed, data are presented as mean ± SD. For non-normally distributed datasets, data are presented as the median with the interquartile range (IQR). For comparisons among more than two groups at a single endpoint, one-way ANOVA was used when assumptions for parametric testing were met; otherwise, the Kruskal–Wallis test was applied. When an overall difference was detected, post-hoc multiple comparisons were performed using Dunnett’s (vs. control) correction as appropriate. For experiments with two independent variables, two-way ANOVA was applied with multiple-comparisons correction. All tests were two-sided, and *P* < 0.05 was considered statistically significant (ns, not significant; * *P* < 0.05; ** *P* < 0.01; *** *P* < 0.001; **** *P* < 0.0001). Details of immunofluorescence image quantification, experimental replication, and extended statistical procedures are provided in the Supplementary Information (Supplementary Methods SM9).

## Results

### Heterogeneity in FAP expression and [^161^ Tb]Tb-FAPI-46 uptake in human PDAC tissues

**Fig. 1:**
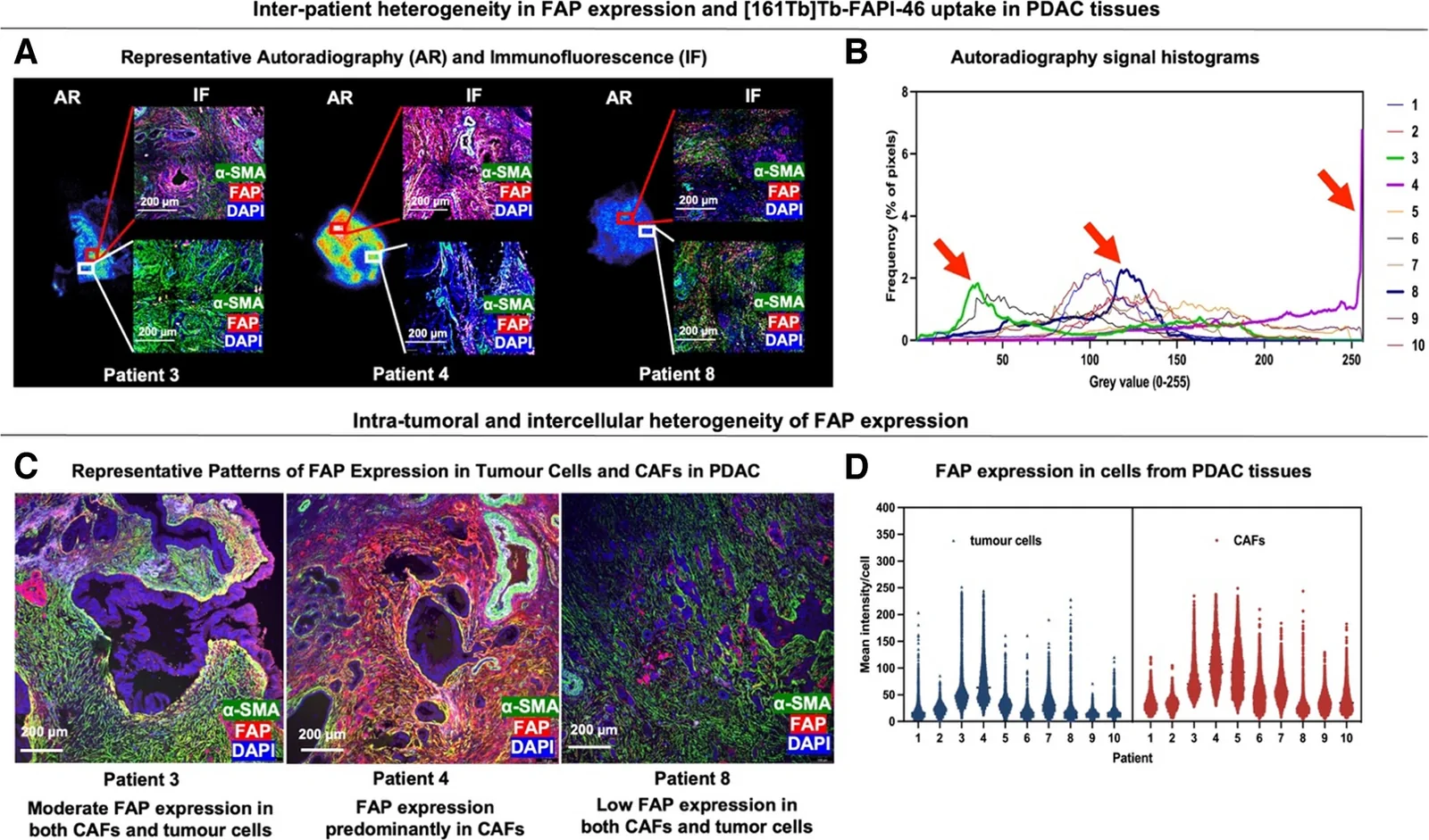
FAP immunofluorescence and [^161^ Tb]Tb-FAPI-46 autoradiography in human PDAC tissues. **A** Representative autoradiography (AR) images of PDAC sections following incubation with [^161^ Tb]Tb-FAPI-46, alongside corresponding immunofluorescence (IF) from the indicated regions (Patients 3, 4, and 8). IF channels: α-SMA (green), FAP (red), and nuclei (DAPI, blue). **B** Pixel-intensity histograms of AR signals for PDAC specimens from Patients 1–10; arrows highlight example features within the distributions. **C** Representative IF images illustrating spatial patterns of FAP staining within tumour and stromal areas in PDAC tissues (Patients 3, 4, and 8). **D** Quantification of FAP fluorescence intensity per cell in tumour cells and CAFs in Patients 1–10 Abbreviations: AR, autoradiography; IF, immunofluorescence; CAFs, cancer-associated fibroblasts; PDAC, pancreatic ductal adenocarcinoma; α-SMA, alpha smooth muscle actin

To better understand the relationship between FAP expression and [^161^ Tb]Tb-FAPI-46 uptake, as well as the heterogeneity of FAP expression in PDAC, we performed immunofluorescence (IF) staining on sections from 10 PDAC patients and carried out autoradiography (AR) using [^161^ Tb]Tb-FAPI-46. In representative matched regions, higher AR hotspots visually coincided with stronger FAP staining, whereas low-uptake areas showed weaker FAP staining (SI Fig. 1, all patients; Fig. 1A, representative images). Notably, regions lacking detectable FAP expression still contained abundant stromal cells and some tumour cells, highlighting that FAP is not uniformly expressed across the stromal compartment in PDAC. To further characterise the spatial scale of uptake non-uniformity, distances between autoradiographic hotspots and low-uptake regions (SI Fig. 1, red and white boxes) were measured in representative tissue sections. To further assess tumour–stroma proximity at the cellular level, we performed an exploratory nearest-neighbour analysis in the same 10 PDAC sections. The median distance from tumour cell to the nearest CAF was 20.1 µm (25th–75th percentile: 17.6–22.7 µm; *n* = 10), indicating close micrometre-scale tumour–stroma proximity. Nevertheless, when characterising the spatial scale of uptake non-uniformity, the median distance between autoradiographic hotspot and coldspot was 7.7 mm (25th–75th percentile: 5.2–10.1 mm; *n* = 10), occurred over substantially larger, millimetre-scale spatial distances. This suggests that regional uptake heterogeneity was not determined solely by tumour–stroma proximity, but may also reflect differences in FAP expression intensity and the local abundance of FAP-expressing stromal cells. Thus, [^161^ Tb]Tb-FAPI-46 uptake patterns varied markedly between patients, ranging from low, patchy signals to strong and more widespread binding (SI Fig. 1, all patients; Fig. 1A, representative images). This interpatient variability was further supported by AR histograms (Fig. 1B). Together, these results highlight substantial interpatient differences in both overall radiopharmaceutical deposition and heterogeneity within tumour tissue.

IF corroborated marked interpatient variability in the relative FAP staining patterns between CAF-rich stroma and tumour regions (Fig. 1C). For example, patient 3 shows moderate FAP signal in both stromal cells (CAFs) and tumour regions, patient 4 displays FAP expression predominantly within the CAF-rich stroma, whereas patient 8 exhibits generally low FAP staining in both compartments (Fig. 1C). Consistently, single-cell–level quantification reveals patient-specific differences in the relative FAP intensity distributions of CAFs and tumour cells, with CAFs overall exhibit higher and more variable FAP expression than tumour cells (Fig. 1D).

Together, these data indicate that FAP concentration in PDAC is heterogeneous at the patient level, varies across regions within the same tumour, and differs substantially between tumour cells and CAFs populations.

### CAF activation, uptake/internalisation, and single-cell nuclear dose estimation at low vs high added radioactivity

To evaluate how stromal activation influences [^161^ Tb]Tb-FAPI-46 binding, normal fibroblasts (NF) were activated to CAF-like cells by exposure to tumour cell (PSN-1) supernatant. IF confirmed elevated FAP signal in CAFs compared with NF, whereas PSN-1 tumour cells displayed minimal FAP staining. Consistent with this expression pattern, cellular uptake of [^161^ Tb]Tb-FAPI-46 scaled with FAP levels (CAFs > NF > PSN-1, *P* < 0.0001), supporting a direct correlation between tracer accumulation and target abundance (SI Fig. 2).

**Fig. 2:**
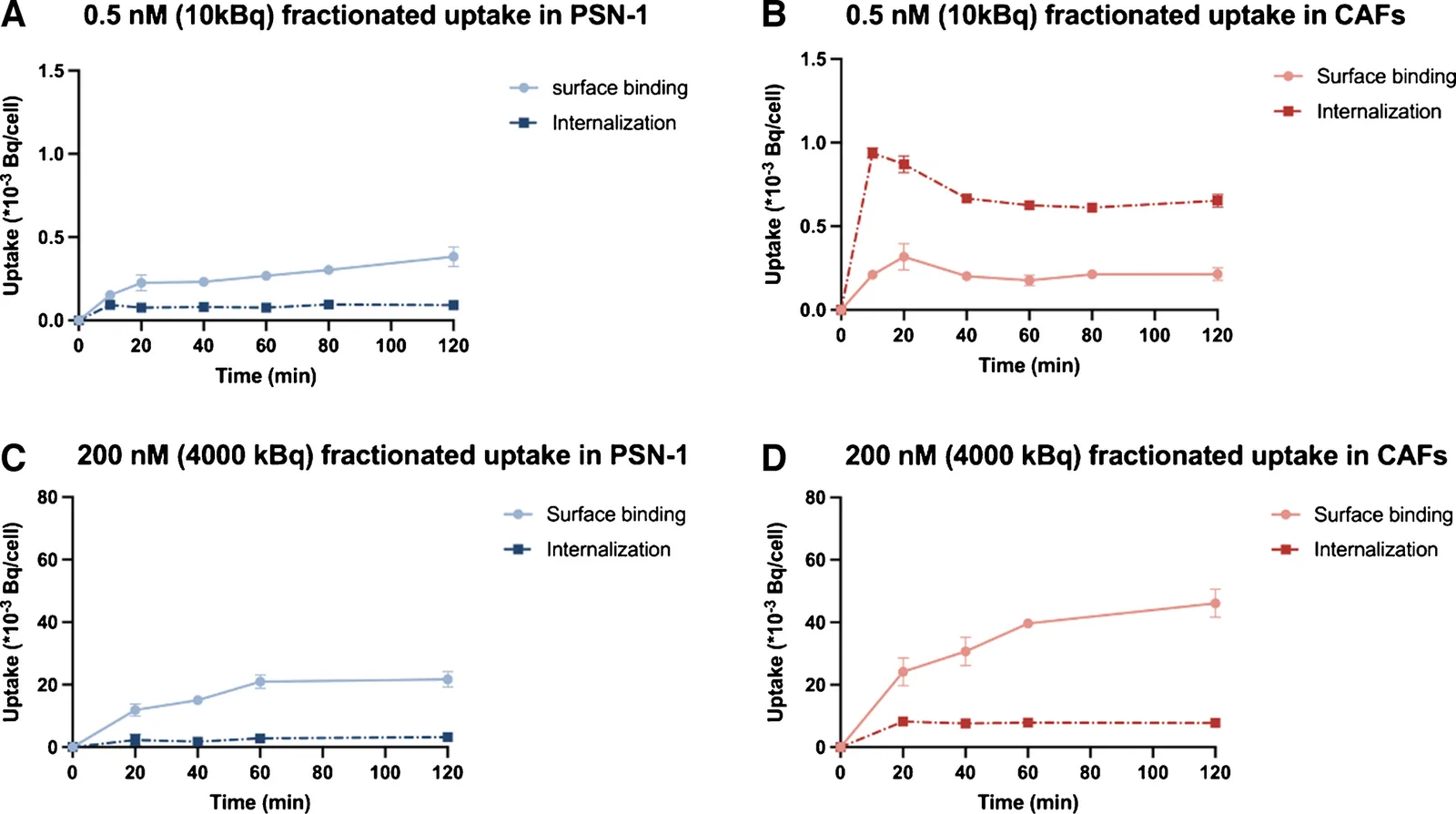
Dose-dependent shift in fractionated uptake of [^161^ Tb]Tb-FAPI-46 between internalisation and surface binding. **A**, **B** Time-dependent fractionated uptake in PSN-1 cells (**A**) and CAFs (**B**) at low ligand concentration (0.5 nM; 10 kBq). **C**, **D** Time-dependent fractionated uptake in PSN-1 cells (**C**) and CAFs (**D**) at high ligand concentration (200 nM; 4000 kBq). In PSN-1 cells (low FAP expression), surface-bound activity predominated over internalised activity at both conditions. In CAFs (high FAP expression), internalisation predominated at 0.5 nM, whereas surface binding predominated at 200 nM

We next assessed how added radioactivity concentration affects fractionated uptake (surface binding vs internalisation) (Fig. 2, SI Fig. 3), with the aim of estimating and comparing the resulting nuclear absorbed dose in CAFs versus PSN-1 at low (0.5 nM, 10 kBq) and high activities (200 nM, 4000 kBq) (SI Table 1). At low added activity amounts, PSN-1 showed low uptake dominated by surface association (Fig. 2A), whereas CAFs exhibited higher uptake with pronounced internalisation (Fig. 2B). However, despite the higher AUC_0–240 min_ in CAFs (8.5 vs 3.4 Bq·s/cell, SI Fig. 3A, Table 1), the estimated nuclear absorbed dose was still higher in PSN-1 than in CAFs, although the difference remained relatively small (7.1 vs 1.7 mGy; SI Table 1). At higher added radioactivity, uptake in both cell types became surface-dominated with reduced internalisation, consistent with receptor saturation (Fig. 2C–D). Notably, although CAFs still showed a larger AUC_0–240 min_ (401.5 vs 184.8 Bq·s/cell, SI Fig. 3B, SI Table 1), the difference in estimated nuclear absorbed dose became much more pronounced, with PSN-1 receiving an approximately tenfold higher dose than CAFs (388 vs 40 mGy; SI Table 1).

FAP uptake at 4000 kBq is largely saturated and is driven predominantly by surface-associated binding in both PSN-1 and CAFs (SI Fig. 3C-D). This shift toward non-specific surface adsorption was evident regardless of FAP expression level, indicating that increasing activity and amount mainly amplifies non-target binding rather than target-mediated internalisation, which may limit therapeutic gain while increasing the risk of off-target exposure. However, in this in vitro study, we still applied the high-activity condition to explore radiobiological effects.

To assess whether cell adhesion status affected uptake, we additionally compared attached and suspended conditions in PSN-1 cells and CAFs. Attached cells showed higher surface-associated [^1^⁶^1^ Tb]Tb-FAPI-46 activity than suspended cells, whereas internalised activity was not significantly different between the two conditions (SI Fig. 3E-F).

### 2D clonogenic survival and DNA damage assessment

**Fig. 3:**
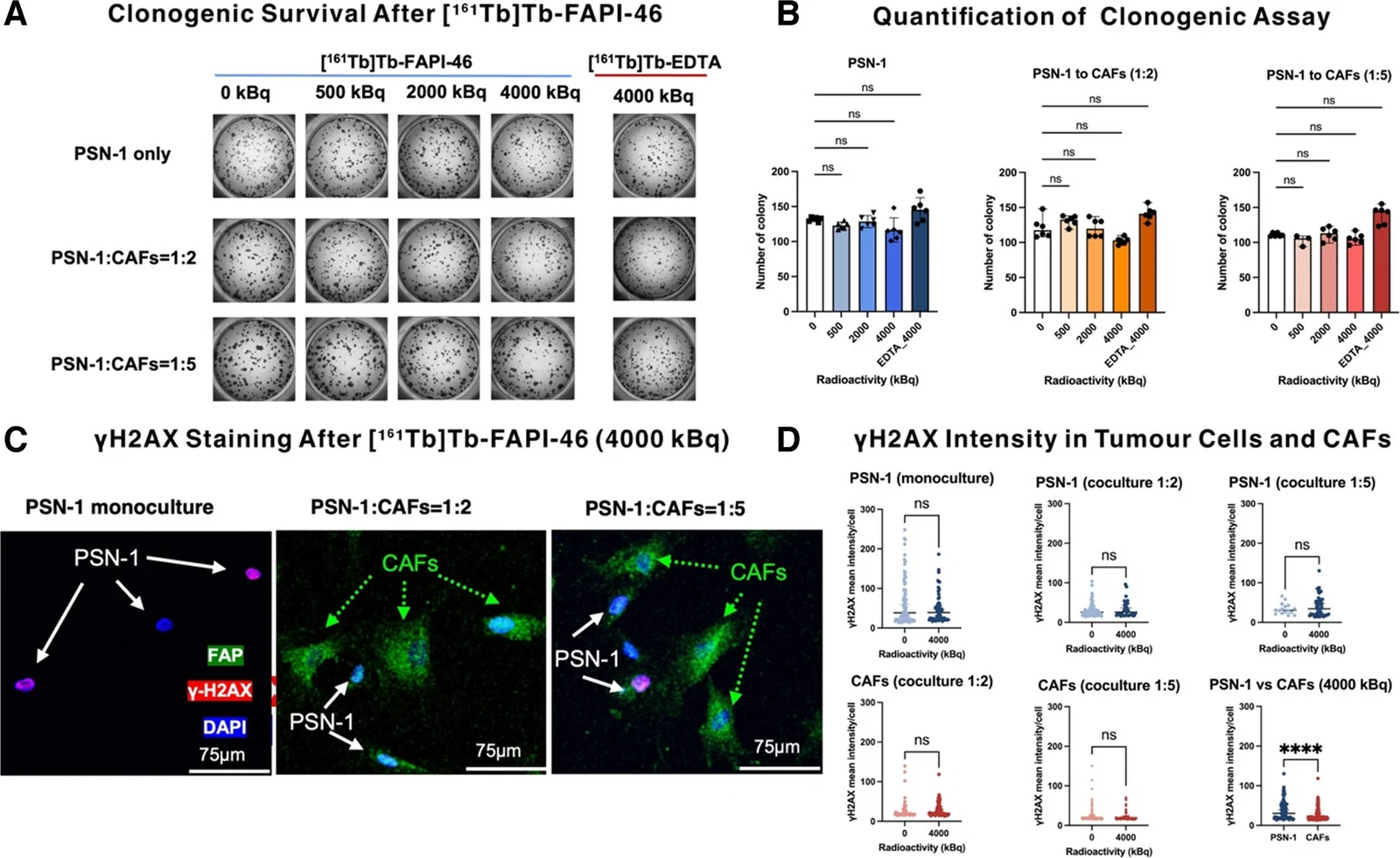
2D clonogenic survival and γH2AX-based DNA damage assessment after [^161^ Tb]Tb-FAPI-46 treatment in PSN-1 and PSN-1/CAF co-cultures. **A** Clonogenic assay images for PSN-1 monoculture and PSN-1/CAF co-cultures (PSN-1:CAFs = 1:2 and 1:5) after exposure to increasing activities of [^161^ Tb]Tb-FAPI-46; [^161^ Tb]Tb-EDTA at 4000 kBq was included as a non-targeted control. **B** Quantification of colony numbers corresponding to (A) across models and activities. **C** Representative immunofluorescence images of γH2AX (DNA damage marker), FAP (marker of CAFs), and nuclei (DAPI) acquired 24 h after treatment (4000 kBq) in PSN-1 monoculture and PSN-1/CAF co-cultures; right panels show a higher-magnification view. **D** Quantification of γH2AX intensity in tumour cells and CAFs under the indicated conditions. Abbreviations: CAFs = cancer-associated fibroblasts; DAPI = 4′,6-diamidino-2-phenylindole; FAP = fibroblast activation protein; γH2AX = phosphorylated histone H2AX

To evaluate the cytotoxic effect of [^161^ Tb]Tb-FAPI-46 in a 2D monolayer setting, clonogenic survival was assessed in PSN-1 monoculture and in PSN-1/CAF co-cultures (PSN-1:CAFs = 1:2 and 1:5) across a dose range up to 4000 kBq. Interestingly, no significant reduction in colony formation was observed in any model at any tested activity (Fig. 3A and B). In addition, the non-targeted control [^161^ Tb]Tb-EDTA did not alter colony numbers, supporting the absence of non-specific radiotoxic effects under these experimental conditions. Overall, these results indicate that [^161^ Tb]Tb-FAPI-46 did not elicit a measurable therapeutic effect in 2D monolayer cultures.

DNA double-strand break signalling was assessed by γH2AX immunofluorescence 24 h after treatment. Consistent with the clonogenic results, γH2AX intensity did not significantly change in PSN-1 cells or CAFs, even at 4000 kBq (Fig. 3C and D); however, γH2AX levels were consistently lower in CAFs than in PSN-1 cells both at baseline (0 kBq) (median [IQR]: 18 [16–21] vs 27 [19–33], *P* < 0.0001) and after treatment (Fig. 3D) (median [IQR]: 19 [16–22] vs 30 [19–53], *P* < 0.0001), suggesting reduced DNA damage signalling in CAFs and potentially greater radioresilience under the tested conditions.

### Therapeutic efficacy in 3D spheroids

**Fig. 4:**
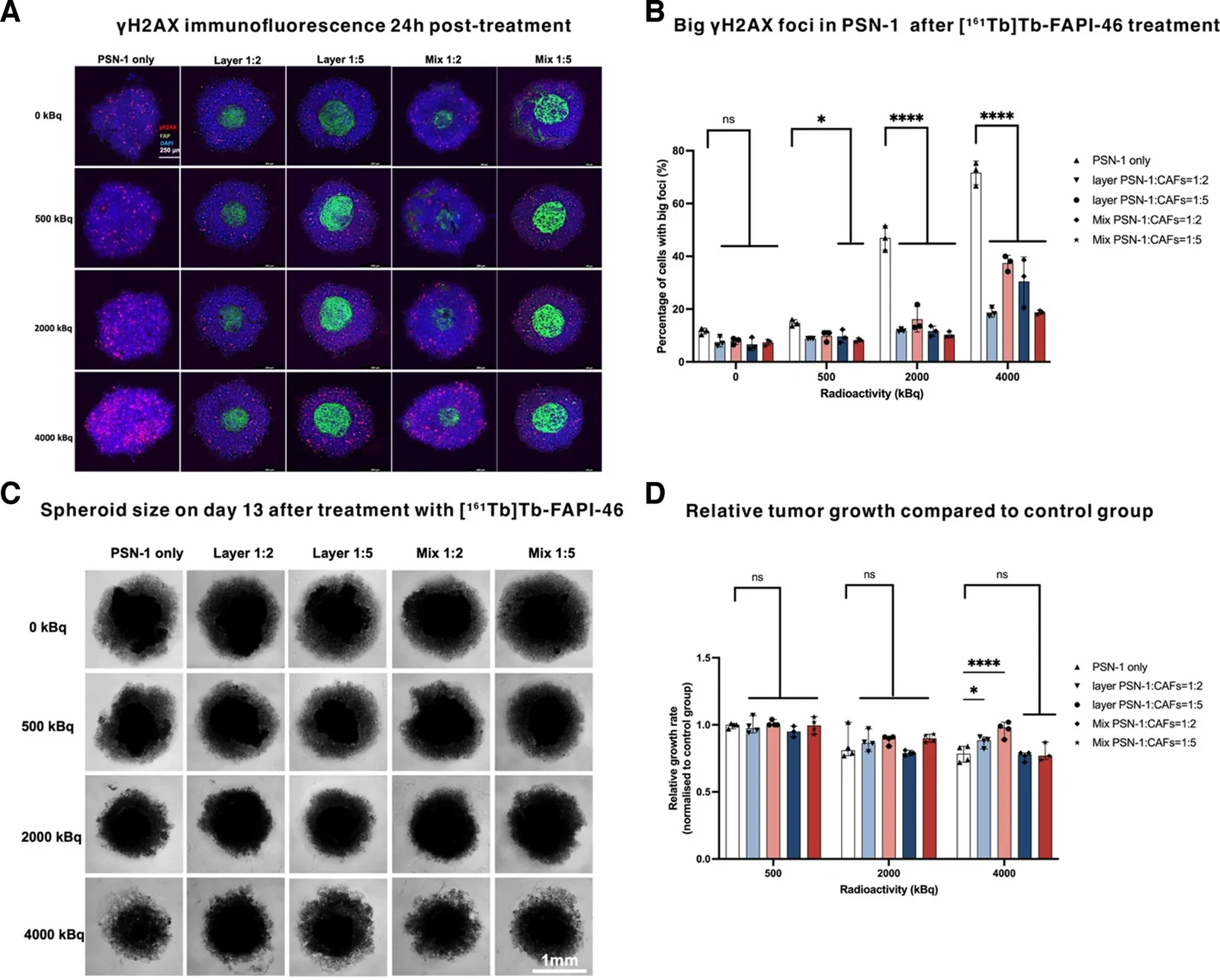
Dose-dependent growth inhibition and DNA damage in 3D spheroids after [^161^ Tb]Tb-FAPI-46 treatment. **A** Representative whole-spheroid immunofluorescence images acquired 24 h post-treatment showing γH2AX (DNA damage marker), FAP, and nuclei (DAPI) across spheroid configurations and activities. **B** Quantification of PSN-1 cells containing large γH2AX foci across spheroid models and activities. CAFs = cancer-associated fibroblasts; DAPI = 4′,6-diamidino-2-phenylindole; FAP = fibroblast activation protein; γH2AX = phosphorylated histone H2AX. **C** Representative brightfield images of spheroids on day 13 after treatment with [^161^ Tb]Tb-FAPI-46 (0–4000 kBq) in PSN-1-only spheroids and PSN-1/CAF spheroids with CAFs arranged as an outer layer (Layer; PSN-1:CAFs = 1:2 or 1:5) or mixed throughout the spheroid (Mix; PSN-1:CAFs = 1:2 or 1:5). **D** Relative spheroid growth normalised to the corresponding untreated control group for each spheroid configuration across activities. Values < 1 indicate growth inhibition relative to the 0 kBq control, whereas higher values indicate weaker inhibition

To better recapitulate the spatial architecture of human tumours, we established 3D spheroids and assessed their response to [^161^ Tb]Tb-FAPI-46 treatment (Fig. 4). Given our prior observation that the distribution of CAFs is heterogeneous in PDAC patient tissues, we generated PSN-1/CAF spheroids with different cell ratios and with two spatial configurations: layered spheroids, generated by sequential seeding to create an intended CAF-shell/tumour-core arrangement, and mixed spheroids, generated by simultaneous seeding of PSN-1 cells and CAFs. Notably, in the layer model, the final organisation differed from the intended “CAF shell” arrangement: CAFs redistributed toward the spheroid core, potentially consistent with a higher tolerance of CAFs to low-oxygen stress [33] (Fig. 4A). This redistribution does not detract from the clinical relevance of the model, as tumour cells and CAFs in PDAC are intermingled and often form mutually enclosing structures. In the mixed model, although a substantial fraction of CAFs also localised to the spheroid centre, CAFs remained detectable in peripheral regions and intermixed with PSN-1 cells (Fig. 4A).

**Fig. 5:**
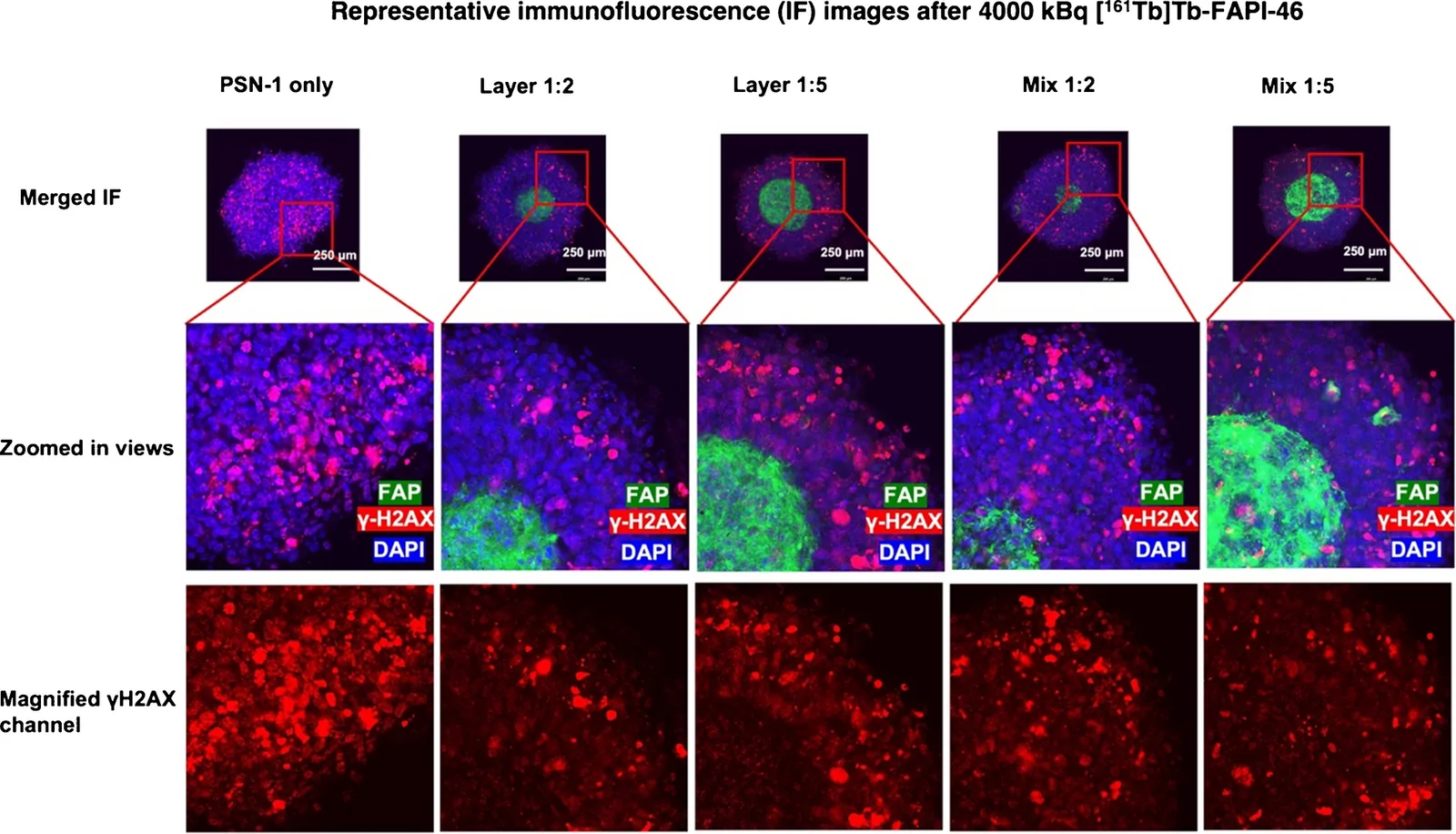
Spatial configuration–dependent γH2AX (DNA damage) patterns after 4000 kBq [^161^ Tb]Tb-FAPI-46. Representative immunofluorescence images of spheroids 24 h after treatment with 4000 kBq [^161^ Tb]Tb-FAPI-46. Merged staining shows γH2AX (red), FAP (green), and DAPI (blue). Red boxes indicate regions selected for magnification. Compared with the layered co-culture models, the mixed co-culture models display a more spatially homogeneous γH2AX signal across the spheroid

To elucidate the contribution of CAFs to FAP-TRT, we compared [^161^ Tb]Tb-FAPI-46 responses between PSN-1 monoculture spheroids and PSN-1/CAF co-culture spheroids by assessing both DNA damage response at the early stage after treatment (24 h) and long-term growth kinetics (day 0-day 13). We first measured γH2AX foci formation in PSN-1 cells at 24 h post-exposure as an early indicator of irradiation-induced DNA double-strand breaks and cellular radiosensitivity to [^161^ Tb]Tb-FAPI-46 (Fig. 4A and B). PSN-1 monoculture spheroids displayed a clear activity-dependent increase in DNA damage, with a pronounced rise at 2000 kBq and an even stronger response at 4000 kBq. In contrast, all PSN-1/CAF co-culture conditions showed markedly lower levels of big γH2AX foci in PSN-1 cells at the same activities, with differences becoming significant from 500 kBq and highly significant at 2000–4000 kBq, indicating that the presence of CAFs attenuates the acute DNA damage burden in tumour cells (Fig. 4A and B). We also compared distribution of γH2AX across spheroid configurations treated with 4000 kBq [^161^ Tb]Tb-FAPI-46 to assess how spatial organisation influences DNA damage response (Fig. 5). In the layered co-culture spheroids, γH2AX signals were more heterogeneous, with reduced signal in the inner regions where CAFs are located, consistent with the presence of a relatively radioresistance of CAFs. In contrast, the mixed co-culture spheroids showed a more uniform γH2AX distribution across the spheroid, including central areas that contain a mixture of PSN-1 and CAFs and are enriched in FAP-positive cells.

We next evaluated the long-term therapeutic consequences of [^161^ Tb]Tb-FAPI-46 by monitoring spheroid growth dynamics over time (Fig. 4C and D), which integrates not only direct radiation effects but also broader radiobiological processes within 3D co-cultures, such as bystander signalling, paracrine interactions, and crossfire effect between tumour cells and CAFs [34, 35]. Compared with the modest effects observed in 2D monolayers, [^161^ Tb]Tb-FAPI-46 elicited notable growth suppression in 3D spheroids, with stronger inhibition generally observed at higher activities across multiple configurations (SI Fig. 4; Fig. 4C). Because baseline growth dynamics differed across model configurations, treatment effects were evaluated using growth ratios normalised to the corresponding control condition (0 kBq) within each model. Our result showed that at 500 kBq, the relative growth rate remained close to 1 in both PSN-1 monoculture and all PSN-1/CAF co-cultures, indicating negligible inhibition and no significant differences between co-culture and monoculture (all *P* > 0.27). At 2000 kBq, relative growth rates fell below 1 in both monoculture and co-culture spheroids, but the extent of suppression was comparable and again did not differ significantly between the groups (all *P* > 0.10). In contrast, at 4000 kBq, a clear divergence emerged: PSN-1-only spheroids and the mixed co-culture models showed relative growth well below 1, indicating robust inhibition, whereas the layered co-cultures displayed significantly higher relative growth rates than the PSN-1-only spheroids (PSN-1:CAF = 1:2, *P* = 0.0188; PSN-1:CAF = 1:5, *P* < 0.0001), while mixed co-culture models did not differ significantly from PSN-1 monocultures (PSN-1:CAF = 1:2, *P* = 0.6940; PSN-1:CAF = 1:5, *P* = 0.7924) (Fig. 4C and D). This pattern suggests that the spatial arrangement of CAFs and tumour cells modulates FAP-TRT efficacy, with mixed architectures potentially enabling a more effective crossfire contribution than layered configurations.

### Conditioned-medium experiment and cytokine profiling

**Fig. 6:**
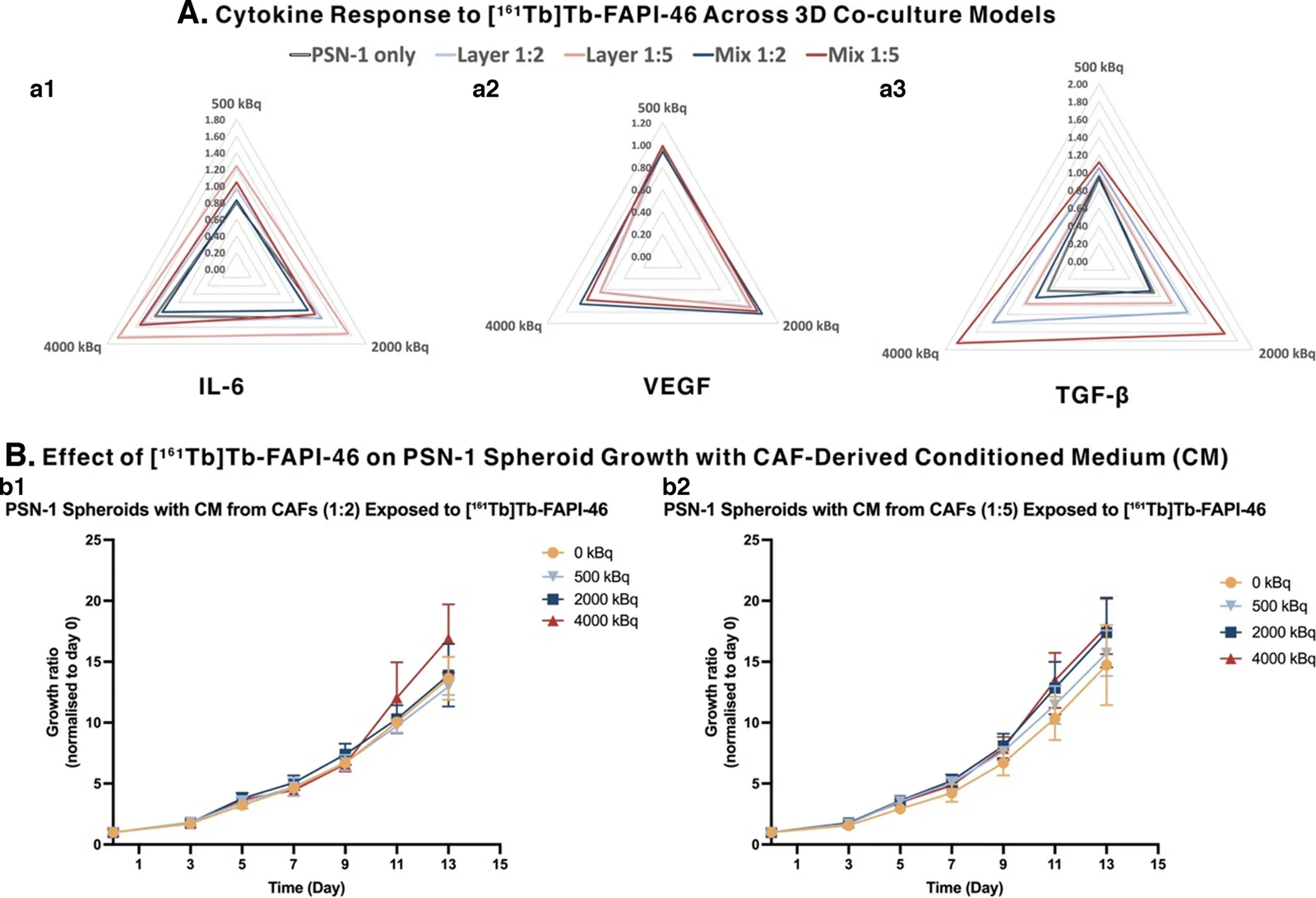
Cytokine profiling and conditioned-medium effects in 3D spheroid models treated with [^161^ Tb]Tb-FAPI-46. **A** Cytokine profiling of culture supernatants collected at day 13 from PSN-1 monoculture spheroids and PSN-1/CAF co-culture spheroids (layer 1:2, layer 1:5, mix 1:2, mix 1:5) treated with [^161^ Tb]Tb-FAPI-46 at 500, 2000, or 4000 kBq (cytokine levels were derived from three replicate wells per condition and are presented as fold change relative to the 0 kBq condition within each corresponding model). Radar plots summarise relative levels of IL-6 (a1), VEGF (a2), and TGF-β (a3) across configurations. **B** Growth kinetics of PSN-1 spheroids cultured with conditioned medium (CM) derived from irradiated CAF cultures corresponding to 1:2 (b1) or 1:5 (b2) settings

To explore potential CAF-mediated mechanisms that may modulate [^161^ Tb]Tb-FAPI-46 efficacy, we performed cytokine profiling using culture supernatants collected at day 13 (Fig. 6A). Across the 3D co-culture models, IL-6 and TGF-β levels tended to increase with higher CAF content and higher radioactivity, whereas VEGF showed comparatively smaller changes across conditions, suggesting the paracrine signalling (cellular interaction between tumour cells and CAFs) during FAP-TRT is shaped by CAF abundance and spatial organisation, and is further modulated by the administered radioactivity.

We next assessed whether CAF-derived soluble factors could influence PSN-1 spheroid growth using a conditioned-medium (CM) from the irradiated CAFs spheroid 3 days after FAP-TRT (Fig. 6B). When PSN-1 spheroids were cultured with CM derived from the 1:2 setting (i.e., irradiated CAFs outnumbering PSN-1 cells by twofold), only the high-activity group (4000 kBq) showed a slight increase in spheroid growth relative to the control condition at the late stage. In contrast, CM derived from the 1:5 setting (fivefold more irradiated CAFs than PSN-1) was associated with a consistent, albeit modest, late-stage growth increase across all radioactivity groups (500, 2000, and 4000 kBq) compared with control. Notably, these trends did not reach statistical significance, but they collectively suggest that CAF secretomes—particularly under higher CAF abundance—may support PSN-1 growth over time.

## Discussion

In this study, we aimed to identify key determinants of FAP-TRT efficacy. We focused on the radiobiological role of naturally FAP-expressing CAFs in FAP-TRT by integrating dose-relevant measurements with biological readouts. By combining PDAC patient tissue profiling with mechanistic 2D/3D co-culture models, we connected cell- and spatial-level heterogeneity in FAP expression and radioligand uptake to quantitative nuclear dose estimates and downstream radiobiological consequences (DNA damage signalling, survival, and longer-term growth control), thereby providing a more actionable framework for optimising FAP-TRT. Notably, our 3D co-culture spheroid system represents one of the few FAP-TRT models based on naturally FAP-expressing fibroblasts co-cultured with tumour cells, providing a well-controlled platform to specifically interrogate the role of CAFs and their interactions with tumour cells in shaping therapeutic response. Collectively, our data suggest that (i) FAP expression and [161 Tb]Tb-FAPI-46 uptake in PDAC patient tissues are spatially heterogeneous, with variable FAP levels across CAFs and tumour cells; (ii) the nuclear dose difference between low-FAP tumour cells and high-FAP CAFs depends on added activity, driven by activity-dependent shifts in uptake/internalisation; (iii) CAFs exhibit greater radioresistance than PSN-1, as indicated by a weaker DNA damage response under high-activity FAP-TRT; and (iv) CAF abundance and spatial configuration modulate both early DNA damage responses and longer-term therapeutic efficacy, potentially through radioligand partitioning, crossfire geometry, and CAF-derived paracrine signalling. Overall, these findings support a dual role for CAFs in FAP-TRT: while CAF-associated activity can contribute to crossfire irradiation of neighbouring tumour cells, CAFs may also attenuate the efficacy of FAP-TRT, potentially through radioligand partitioning towards the stromal compartment and CAF-derived paracrine signalling that promotes tumour growth.

FAP expression in PDAC is heterogeneous at multiple levels: it differs between patients, varies across regions within the same tumour, and can diverge substantially between tumour cells and CAF populations. Importantly, in contrast to studies emphasising FAP as a CAF-enriched stromal target [36, 37], FAP-TRT is therefore commonly interpreted as acting mainly through CAF targeting [38]. Our data support the possibility that tumour cells in PDAC may also express FAP, implying that the distribution of FAP-expressing cells is more complex than a purely stromal pattern, which is consistent with prior reports of FAP expression in both PDAC tumour cells and CAFs [4, 5]. PDAC tissue sections revealed pronounced intratumoral heterogeneity in [^161^ Tb]Tb-FAPI-46 uptake, suggesting that activity concentrated in “hot spots” may provide insufficient cross-irradiation to adjacent low-uptake (“cold”) regions that remain densely cellular. Consistent with this concept, Falzone et al. [39] and Feijtel et al. [40] showed that spatial heterogeneity in radionuclide distribution at subcellular and multicellular scales is closely linked to biological effect and tumour control probability. Together, these findings support the notion that FAP-TRT efficacy is influenced by the spatial distribution of FAP-expressing cells across both micro- (cellular/subcellular) and macro-scale (regional) tumour compartments. Clinically, these findings argue for more nuanced patient selection and stratification for FAP-TRT. Assessment of FAP abundance, regional uptake heterogeneity, and the proximity between FAP-positive and FAP-low compartments may help identify tumours in which crossfire irradiation is likely to cover relevant tumour regions. However, because FAP-TRT represents a stromal-targeted rather than purely tumour-cell-directed strategy, uptake intensity alone is unlikely to be sufficient as a predictive biomarker. Future stratification may also need to consider the radiobiological context of stromal targeting, including CAF radioresponsiveness, CAF–tumour spatial organisation, CAF subtype composition, and CAF-related paracrine signatures. These considerations may further support rational combination strategies, for example, combining FAP-TRT with inhibitors of TGF-β or IL-6/JAK–STAT signalling, although this requires dedicated preclinical validation.

Although FAP was detectable in tumour cells, CAF-associated staining was markedly stronger in most patients, leading us to hypothesise that CAFs are the primary determinants of FAP-targeted radioligand uptake and microdosimetry in PDAC. We therefore used 2D and 3D co-culture models, including mixed configurations in which FAP-high CAFs coexist with PSN-1 tumour cells expressing relatively low FAP, to evaluate the impact of CAF abundance and spatial organisation on therapeutic outcome. At low added radioactivity, PSN-1 exhibited low overall uptake that was dominated by surface-associated binding with only a minor internalised fraction, whereas CAFs showed substantially higher uptake with a more pronounced internalisation component, which is aligned with FAP expression level. In contrast, at high added radioactivity, uptake in both PSN-1 and CAFs became predominantly surface-associated while the relative internalised fraction decreased. This pattern is consistent with findings in PSMA-targeted systems, where changing the applied activity amount alters the partitioning between surface-bound and internalised activity [41]. Even though CAFs exhibited a larger cumulated activity (AUC) than PSN-1 at high added activity, single-cell dosimetry predicted a substantially higher nuclear absorbed dose in PSN-1. This divergence reflects that nuclear dose is determined not only by cumulated activity, but also by cell/nuclear geometry and subcellular activity distribution, which together define the nucleus S value [42, 43]. Accordingly, the magnitude of uptake, at scales accessible by PET or SPECT imaging, alone is an insufficient surrogate for nuclear dose, and activity-dependent shifts in internalisation and compartmentalisation can markedly alter the dose delivered to the nucleus. Our additional comparison between attached and suspended cells further showed that adhesion status mainly affected surface-associated [^1^⁶^1^ Tb]Tb-FAPI-46 binding, whereas internalised activity was not significantly altered. The relative pattern of higher uptake/internalisation in CAFs than in PSN-1 cells was maintained, suggesting that adherent 2D uptake assays remain useful for comparing cell-type-specific uptake behaviour, but should not be interpreted as a direct quantitative surrogate for uptake during suspension-based clonogenic exposure. In this context, the limited radiobiological effects observed in 2D models are consistent with the microdosimetric considerations described above. This highlights the limitations of 2D culture systems for evaluating FAP-TRT, where spatial organisation and crossfire effects are critical determinants of efficacy.

In 3D monoculture spheroids, PSN-1 showed a clear activity-dependent DNA damage response, whereas in PSN-1/CAF co-culture spheroids, PSN-1 exhibited markedly fewer big γH2AX foci at matched activities. These results suggest that CAFs reduce the acute DNA damage burden experienced by tumour cells in early response windows. A potential, but inferential, explanation is a “radioligand sink” effect: because CAFs demonstrate higher uptake, they may sequester a larger fraction of [^161^ Tb]Tb-FAPI-46, thereby reducing the effective activity available to tumour cells and shifting absorbed dose partitioning toward stroma. Beyond CAF presence, architecture also shaped the spatial DNA damage response. In co-culture spheroids, layered configurations tended to show more heterogeneous γH2AX patterns with relatively weaker signals in inner regions. In contrast, mixed co-culture spheroids displayed a more spatially homogeneous γH2AX distribution across the spheroid, including central regions containing intermixed PSN-1 and FAP-positive CAFs. In an in silico tumour–stroma model, Tranel et al. explicitly compared absorbed-dose distributions when either tumour cells or CAFs were treated as the dominant “source” compartment and showed that the relative dose delivered to tumour targets depends strongly on the spatial separation between tumour clusters and CAFs and on radionuclide range—i.e., CAF-localised activity can leave parts of the tumour underdosed as distances increase, thereby increasing dose heterogeneity [44]. Consistently, our long-term spheroid assays showed strong growth inhibition in PSN-1–only and mixed co-cultures (relative growth < 1), whereas layered co-cultures exhibited significantly higher relative growth rates than PSN-1–only spheroids, suggesting attenuated treatment efficacy in the layered configuration. Overall, this pattern suggests that the spatial arrangement of CAFs and tumour cells modulates FAP-TRT efficacy, with mixed architectures potentially enabling a more effective crossfire contribution than layered configurations.

Despite the clear influence of spatial organisation and crossfire on treatment efficacy, these physical factors alone may not fully explain the divergent long-term growth patterns observed across co-culture configurations. We therefore next examined whether irradiated CAFs could remodel the tumour microenvironment through paracrine signalling under FAP-TRT. IL-6, TGF-β, and VEGF were selected as representative CAF-related paracrine mediators covering three major tumour microenvironmental processes in PDAC: inflammatory signalling, stromal activation, and angiogenic remodelling, respectively [45–47]. Cytokine profiling across our 3D co-culture spheroids suggested a CAF-driven paracrine signature during FAP-TRT: IL-6 and TGF-β levels were consistently higher in CAF-containing models than in monocultures across activity conditions, whereas VEGF showed comparatively modest changes. This pattern aligns with prior PDAC literature describing CAFs program enriched for inflammatory cytokines such as IL-6 and prominent TGF-β signalling in stromal activation states, which could promote tumour growth [48, 49]. CM experiments further suggested that CAF-derived soluble factors can promote PSN-1 spheroid growth when supernatant from irradiated CAF spheroids (collected 3 days after treatment) was applied, with trends that did not reach statistical significance but collectively point to a potential growth-supporting CAF secretome, particularly at higher CAF abundance. Notably, the timing of supernatant collection may limit interpretation: sampling at a single early time point might not capture the evolving dynamics of CAF–tumour interaction and delayed secretory phenotypes.

This study has its limitations. First, our CAF model was generated by conditioning fibroblasts with tumour-cell–derived supernatants rather than using patient-derived primary CAFs. Although this approach yields an activated, FAP-expressing fibroblast phenotype and enables controlled co-culture experiments, it may not fully capture the phenotypic diversity and microenvironmental context of CAF populations in PDAC patients. In addition, because PDAC was selected as a prototypical desmoplastic, CAF-rich model, these findings should not be directly generalised to all tumour types. Their applicability is likely to depend on tumour-specific stromal abundance, FAP-positive CAF distribution, tumour–stroma proximity, CAF subtype composition, and the relative contribution of crossfire irradiation and CAF-mediated paracrine effects. Second, our in vitro models lack immune, vascular, and pharmacokinetic constraints that influence radioligand delivery and microenvironmental feedback in vivo. However, the simplified model employed here allowed easier isolation of parameters and identification of potentially impactful parameters. Third, the intended spatial architecture of the 3D co-culture spheroids was not fully preserved over time. CAFs tended to migrate toward and accumulate in the spheroid core in both the layered and mixed configurations [33]. As a result, CAF and PSN-1 distributions in the mixed model were less uniformly intermingled than originally designed, although a small fraction of CAFs remained at the periphery. Fourth, cytokine measurements were performed on bulk supernatants collected at day 13, which provides only an averaged, endpoint snapshot, only of selected secreted factors, across each spheroid. This approach may mask configuration-dependent differences and spatially restricted cytokine microgradients within 3D co-cultures. Fifth, uptake, internalisation, and efflux assays were performed using adherent 2D cell cultures to compare the relative [^1^⁶^1^ Tb]Tb-FAPI-46 binding and retention patterns between PSN-1 tumour cells and CAFs under controlled conditions. These data should therefore not be interpreted as a direct quantitative surrogate for radioligand uptake during suspension-based clonogenic exposure or for single-cell uptake patterns within 3D spheroids, particularly in mixed tumour cell–CAF models where ligand penetration, spatial organisation, and cell–cell interactions may influence uptake. A further methodological consideration is that different incubation times were used for the 2D clonogenic and 3D spheroid therapy assays. Cells in the clonogenic assay were exposed to [^1^⁶^1^ Tb]Tb-FAPI-46 for 1 h in suspension, whereas spheroids were incubated for 2 h to allow radioligand penetration and binding within the compact 3D structure. This difference may influence cell-associated activity and should be considered when comparing 2D and 3D treatment responses. However, the distinct outcomes between these assays are also likely to reflect differences in biological endpoints, 3D tumour–CAF architecture, ligand penetration, and crossfire irradiation. Finally, absorbed dose partitioning between CAFs and tumour cells was inferred from biological readouts rather than directly quantified. Future studies should integrate microautoradiography with compartment-resolved dosimetry and spatial quantification of radioligand microdistribution, together with time-resolved secretome profiling, to define when CAFs primarily enhance tumour irradiation via crossfire versus when CAF-driven paracrine/bystander signalling becomes the dominant determinant of response. To improve translational relevance, these analyses could be performed in more physiologic platforms—such as vascularized and immune-competent organoids or organ-on-chip systems incorporating perfusion, endothelial barriers, and immune–stromal interactions. In this context, the secretome profiling findings may also help identify cytokine pathways, such as IL-6 and TGF-β signalling, that could be explored as targets for future combination strategies with FAP-targeted radionuclide therapy.

Taken together, our findings indicate that FAP-expressing CAFs play a dual and context-dependent role in FAP-targeted radionuclide therapy. On one hand, CAFs with high radioligand uptake can act as effective radiation sources, enhancing crossfire irradiation and contributing to absorbed dose delivery to neighbouring tumour cells. On the other hand, the high-affinity uptake by CAFs may sequester a substantial fraction of FAP-targeted radiopharmaceuticals, thereby reducing effective activity available to tumour cells and shifting dose partitioning toward the stromal compartment. In parallel, CAFs exhibited a relatively radioresistant phenotype and, following irradiation, were capable of promoting tumour growth through paracrine signalling. Therefore, these observations underscore that CAFs are not merely passive targets of FAP-TRT, but active determinants of both dose distribution and downstream radiobiological outcomes.

## Conclusion

In conclusion, this study demonstrates that the radiobiological impact of FAP-TRT is strongly shaped by the presence, abundance, and spatial organisation of FAP-expressing CAFs. By integrating patient tissue analysis with mechanistic 2D and 3D mono- and co-culture models, we show that CAFs exert a dual influence: they can enhance therapeutic efficacy through crossfire-mediated dose redistribution, while simultaneously limiting tumour-cell dose delivery and promoting tumour regrowth via radioresistance and paracrine signalling. These findings highlight the importance of understanding CAF radiobiology when interpreting FAP-TRT outcomes. Improved characterisation of stromal composition, spatial FAP expression patterns, and CAF-driven biological responses may therefore be critical for optimising FAP-TRT and for refining patient stratification strategies to identify those most likely to benefit from this therapeutic approach.

## Supplementary Information

Below is the link to the electronic supplementary material.

Supplementary file1 (DOCX 2232 KB)

## Data Availability

The datasets generated during and/or analysed during the current study are available from the corresponding author upon reasonable request.
