# Supplementary material for: Context-dependent efficacy of fibroblast activation protein-targeted radionuclide therapy (FAP-TRT): the dual role of CAFs
Source: Eur J Nucl Med Mol Imaging. 2026 Jul 29;53(12):7313–26. doi: 10.1007/s00259-026-08099-4 (PMC13633335; doi:10.1007/s00259-026-08099-4)
Supplement: Supplementary file 1 [file 259_2026_8099_MOESM1_ESM.docx]

**Supplementary Information**

**Supplementary Methods (SM)**

**SM1.** **Immunofluorescence staining, image analysis, and autoradiography of pancreatic ductal adenocarcinoma (PDAC) sections**

Formalin-fixed paraffin-embedded (FFPE) pancreatic ductal adenocarcinoma (PDAC) tissue sections (n = 10) were deparaffinised in xylene (10 min, followed by fresh xylene for 5 min) and rehydrated through graded ethanol (100%, 96%, and 70%; 5 min each), rinsed in distilled water (5 min), and washed twice in PBS (5 min each). Heat-induced antigen retrieval was performed in Tris–EDTA buffer (10 mM Tris, 1 mM EDTA, pH 9.0) by microwave heating for 8 min at 80% power, followed by cooling at room temperature for 15 min and additional cooling under running tap water for 10 min. Slides were washed in PBS (3 × 5 min) and sections were circled with a hydrophobic barrier pen. Sections were blocked and permeabilised for 1 h at room temperature in PBS containing 5% BSA, and 0.1% Triton X-100. Primary antibodies were diluted in blocking solution and incubated overnight at 4°C in a humidified chamber: rabbit anti-FAP (Cell Signaling Technology, #66562; 1:250) and mouse anti-α-smooth muscle actin (α-SMA; clone 1A4; BioLegend, #614852; 1:250). After washing in PBS (3 × 5 min), sections were incubated with fluorophore-conjugated secondary antibodies for 1 h at room temperature protected from light: donkey anti-rabbit Alexa Fluor 555 (Abcam, ab150074; 1:500) and goat anti-mouse Alexa Fluor 488 (Abcam, ab150113; 1:500). Slides were rinsed in PBST (PBS + 0.5% Triton X-100) and washed (3 × 5 min), mounted with ProLong Diamond Antifade Mountant with DAPI, coverslipped, sealed with nail polish and stored at 4°C protected from light prior to acquisition of confocal micrographs (Leica SP8).

Quantitative image analysis of PDAC immunofluorescence sections was performed using QuPath software (version 0.5.0). Regions of interest (ROIs) were manually annotated on the basis of tissue morphology, and vascular structures were excluded from the analysis to avoid misclassification, as vascular/perivascular smooth muscle cells also express α-SMA. Cell detection was performed in QuPath using the DAPI channel to identify individual nuclei. α-SMA was used as a marker of CAF-rich stromal cells, as α-SMA is commonly used to identify activated fibroblasts/myofibroblastic CAFs in PDAC [1], whereas tumour epithelial cells in these sections were considered α-SMA-negative. After cell detection, cells within the annotated ROIs were classified according to α-SMA expression: α-SMA-positive cells were classified as CAFs, whereas α-SMA-negative cells within tumour-rich regions were classified as tumour cells. The resulting classification was visually reviewed and adjusted where necessary based on cellular morphology and tissue architecture. PDAC tumour epithelial cells were identified by their epithelial morphology, including cohesive growth patterns, gland-like or nest-like structures, and relatively round to irregular nuclei. In contrast, CAFs/activated fibroblasts typically showed elongated, spindle-shaped or fibroblast-like morphology and were located within stromal/desmoplastic regions. FAP expression was then quantified separately in the classified CAF and tumour-cell compartments by measuring the mean FAP fluorescence intensity per cell.

Autoradiography was performed on paraffin-embedded tissue sections as described above. After deparaffinising as above, sections were incubated with [^161^Tb]Tb-FAPI-46 (50 kBq/mL) diluted in 10 mM HEPES buffer (pH 7.4) for 1 h on ice in a humidified chamber. After gentle rinsing with ice-cold HEPES buffer (5 times) followed by ice-cold PBS, slides were air-dried. Dried slides were kept for 24 h, then placed in close contact with a phosphor imaging screen and exposed for 90 min in a light-tight cassette. Phosphor screens were scanned using a GE Healthcare Amersham Typhoon autoradiography system.

**SM. 2 Cell Culture and CAF Activation**

**SM. 2.1 Cell culture**

PSN-1 PDAC cells and primary human fibroblasts (VH-25) were used in this study. The VH-25 fibroblast line (derived from a healthy donor) was kindly provided by Prof. Rob Coppes (Section of Molecular Cell Biology, University of Groningen, Groningen, The Netherlands). PSN-1 cells were maintained in RPMI 1640 medium supplemented with 10% foetal bovine serum (FBS), 1% (v/v) L-glutamine, and 1% (v/v) penicillin–streptomycin (100× stock; final 1×). VH-25 fibroblasts were cultured in DMEM supplemented with 15% FBS and 1% (v/v) penicillin–streptomycin (100× stock; final 1×). All cells were cultured at 37°C in a humidified incubator with 5% CO₂. Cells were used within <30 passages after thawing and were routinely tested for mycoplasma contamination using a luminescence-based assay. PSN-1 cell identity was confirmed by short tandem repeat (STR) profiling.

**SM. 2.2 Activation of normal fibroblasts into CAF-like cells using PSN-1 conditioned medium**

PSN-1 cells (~7 × 10^6^ cells) were seeded in T175 flasks and cultured for at least 48 h. When cultures reached ~80% confluency, the supernatant was collected as conditioned medium (CM) and clarified by centrifugation to remove cellular debris. The clarified PSN-1 CM was mixed 1:1 with fresh complete DMEM (50% v/v CM) [2, 3] and applied to VH-25 fibroblasts. VH-25 cells were maintained in CM-containing medium for 4 days to induce CAF-like activation.

**SM. 3 Radioligand synthesis**

Non–carrier-added [¹⁶¹Tb]TbCl₃ (radionuclidic purity ≥99.9%) in 0.05 M HCl was purchased from Terthera B.V. (The Netherlands) in a total volume of 100 µL. For radiolabelling, [¹⁶¹Tb]TbCl₃ (180 MBq, 50 µL, 0.05 M HCl) was added to 100–150 µL of Chelex-treated sodium acetate buffer (0.5 M, pH 4.5) containing 5 nmol of FAPi-46 (Cat. No. HY-137331; MedChemExpress), yielding a final reaction volume of 150 µL. The reaction mixture was heated at 95°C for 10 min. Radiochemical purity (RCP) was determined by radio–instant thin-layer chromatography (radio-iTLC) using iTLC-SG strips (Agilent) and 0.1 M citrate buffer (pH 5.0) as the mobile phase. [^161^Tb]Tb-FAPi-46 was obtained with a radiochemical purity of 98.73 ± 0.70% (n = 6). The iTLC strips were analysed using a phosphor screen imager (Cyclone Plus; PerkinElmer). Prior to use, the final product solution was adjusted to pH 6.5–7.0 with 0.5 M sodium carbonate.

**SM. 4** **In vitro uptake, internalisation, and specificity (blocking), Efflux (cellular retention)**

Cells were seeded in 24-well plates at 1 × 10^5^ cells/well and cultured for 24 h to reach ~80–90% confluency. On the day of the experiment, medium was removed and cells were washed with ice-cold PBS. Cells were then incubated with [^161^Tb]Tb-FAPI-46 at 10 kBq/500 μl/well (low dose) or 4000 kBq/500 μl/well (high dose) at 37°C for the indicated time points (20-120 min). For specificity (blocking) experiments, cells were incubated for 20 min at 37°C under otherwise identical conditions, either in the absence of competitor (total binding) or in the presence of a 100-fold molar excess of unlabelled FAPI-46 (blocking). Cellular association was determined after removing growth medium and washing cells twice with 1 mL PBS. To distinguish surface-bound from internalised activity, cells were incubated with glycine-HCl (1 M in PBS, pH 2.2) for 10 min at room temperature to strip surface-bound radioactivity [4, 5], followed by an additional PBS wash. Cells were lysed using NaOH (1 M, 500 μL/well) to quantify the internalised fraction. Radioactivity was measured using an automatic γ-counter (WIZARD 1470, PerkinElmer). For comparison of adherent and suspension conditions, cells were detached and incubated with [¹⁶¹Tb]Tb-FAPI-46 in suspension under the same activity, volume, temperature, and incubation time as the adherent-cell condition. After incubation, suspended cells were pelleted by centrifugation and washed twice with PBS to remove unbound radioligand. Surface-bound and internalised fractions were then determined using the same glycine-HCl acid-wash and NaOH lysis procedures as described above.

For efflux studies, cells were first incubated with [^161^Tb]Tb-FAPI-46 for 20 min (at 10 kBq/well or 4000 kBq/well). The radioactive medium was then removed, the cells washed, and provided with fresh growth medium. Cells were incubated for additional time points (20 min, 40 min, 60 min, 80 min, 100 min, 120 min) before washing and lysis to determine the remaining cell-associated activity.

**SM. 5 Clonogenic survival (colony formation) assay**

PSN-1 cells were prepared either as monocultures or co-cultures with CAFs at PSN-1:CAF ratios of **1:2** or **1:5**. For each condition, 10000 PSN-1 cells were aliquoted into microcentrifuge tubes; for co-culture conditions, CAFs were added at the corresponding ratios (i.e., 20000 or 50000 CAFs, respectively). Cell suspensions were exposed to **[^161^Tb]Tb-FAPI-46** (0, 500, 2000, or 4000 kBq) in microcentrifuge tubes in fresh complete RPMI 1640 with a total volume of **500 µL** and incubated at **37°C for 1 h**. Following exposure, an aliquot corresponding to one-tenth of the total volume (50 µL) was transferred to 6-well plates, resulting in 1000 PSN-1 cells per well (with the corresponding number of CAFs according to the specified ratios). Fresh complete RPMI 1640 medium was then added to each well to a final volume of 2 mL, thereby diluting residual radioactivity and effectively terminating exposure, as previously described by Chan et al [6]. Cells were plated in triplicate per condition. Medium was gently replaced 24 h after seeding, and cultures were maintained for 7 days to allow colony formation, followed by crystal violet staining and enumeration of colonies containing >50 cells.

**SM. 6 3D Spheroid Models and Radionuclide Treatment**

**SM. 6.1 Spheroid formation**

Spheroids were generated using Nunclon™ Sphera™ 96-well, Nunclon Sphera-treated, U-shaped-bottom microplates (Thermo Scientific; Cat. No. 174929), with one spheroid formed per well. Five spheroid models were established using a constant number of PSN-1 cells (2000 cells/well) and tumour-to-CAF ratios of 1:0, 1:2, or 1:5, at a final volume of 200 µL medium per well across all models:

1. PSN-1 monoculture: 2000 PSN-1 cells/well.
2. Layered 1:2: 2000 PSN-1 cells/well; CAFs added 24 h later.
3. Layered 1:5: 2000 PSN-1 cells/well; CAFs added 24 h later.
4. Mixed 1:2: 2000 PSN-1 + 4000 CAFs/well, co-seeded.
5. Mixed 1:5: 2000 PSN-1 + 10000 CAFs/well, co-seeded.

For **PSN-1 monoculture** spheroids, **2000 PSN-1 cells/well** were seeded **48 h prior to treatment (Day −2)**. For mixed spheroids, PSN-1 cells and CAFs were co-seeded 48 h before treatment (Day −2). For layered spheroids, PSN-1 cells were seeded 48 h before treatment (Day −2) to allow initial spheroid formation; CAFs were added 24 h before treatment (Day −1), and spheroids were cultured for an additional 24 h so that all models underwent a total of 48 h of spheroid development prior to treatment. Immediately after seeding, the plates were centrifuged for 5 min at 1500 rpm to promote cell aggregation and spheroid formation.

**SM. 6.2 Radionuclide therapy**

On day 0, spheroids were exposed to [^161^Tb]Tb-FAPI-46 (0, 500, 2000, or 4000 kBq/well) in complete growth medium (120 µL/well). Spheroids were incubated for 2 h at 37°C to allow sufficient radioligand penetration and binding within the compact 3D spheroid structure, after which the supernatant was removed and spheroids were washed three times with complete growth medium. Fresh complete growth medium (200 µL/well) was then added, and spheroids were returned to incubate.

**SM. 6.3 Long-term monitoring**

Spheroids were cultured for up to 14 days (day 0 - day 13). No medium replacement was performed during the 13-day monitoring period. The outermost wells of the 96-well plates were left empty and were not used for spheroid culture to minimise edge effects caused by medium evaporation. Brightfield images were acquired every other day using a **Thermo Fisher Scientific EVOS XL Core Imaging System** (inverted phase-contrast microscope) to monitor spheroid growth and morphological changes. Spheroid size was quantified by measuring **spheroid cross-sectional area** and **normalised to its area on day 0**.

**SM. 7 DNA Damage Assessment by IF**

**SM. 7.1 IF of 2D**

Immunofluorescence (IF) staining was performed to assess (i) FAP expression across PSN-1, normal fibroblasts (NF), and CAFs, and (ii) FAP and DNA damage (γH2AX) under conditions matching the clonogenic assay. For FAP-only staining, cells were seeded on 8-well chamber slides and cultured for at least 24 h. Cells were washed with PBS and fixed with 4% formaldehyde (in PBS, pH 7.4) for 15 min at room temperature, followed by three PBST (0.5% Triton X-100 in PBS) washes (5 min each). Samples were blocked with PBS-BT (PBS + 5% BSA + 0.1% Triton X-100) for 1 h at room temperature and incubated with an anti-FAP primary antibody (FAP E1V9V rabbit mAb, Cell Signaling Technology #66562; 1:250 in blocking buffer) overnight at 4 °C. After three PBST washes, cells were incubated with donkey anti-rabbit IgG H&L (Alexa Fluor® 555) (Abcam ab150074; 1:500) for 1 h at room temperature in the dark, followed by three PBST washes. Slides were mounted with ProLong™ Diamond Antifade Mountant with DAPI (Invitrogen, Thermo Fisher Scientific; P36962).

For combined FAP/γH2AX staining, additional replicate wells were prepared in parallel to the clonogenic assay for each model at 0 kBq (vehicle) and 4000 kBq [^161^Tb]Tb-FAPI-46 (three extra wells per condition) with sterile coverslips placed in the wells prior to seeding. All steps followed the clonogenic workflow, and coverslips were collected 24 h post-treatment for IF staining. Coverslips were processed as above and co-incubated with anti-FAP (CST #66562; 1:250) and anti-γH2AX (phospho S139) (Abcam ab26350; 1:250) overnight at 4 °C, followed by secondary antibodies (each 1:500): donkey anti-rabbit Alexa Fluor® 555 (Abcam ab150074) and goat anti-mouse Alexa Fluor® 488 (Abcam ab150113). After washing, samples were mounted with ProLong™ Diamond Antifade Mountant with DAPI (P36962). Fluorescence images were acquired using a Leica TCS SP8 confocal microscope.

**SM. 7.2 IF of 3D spheroids**

At 24 h post treatment, spheroids were collected and washed twice in PBS and fixed in 4% paraformaldehyde at 4°C overnight. Fixed spheroids were transferred to PBS and subjected to stepwise dehydration/permeabilisation using a methanol series (10 min each): 50% methanol (in PBS), 80% methanol (in deionised water), and 100% methanol. Samples were then rehydrated and further permeabilised (10 min each) through 20% DMSO/methanol, 80% methanol, 50% methanol, 100% PBS, and finally PBS containing 2% Triton X-100. Spheroids were incubated in penetration buffer (PBS with 0.2% Triton X-100, 0.3 M glycine, and 20% DMSO) for 30 min with gentle shaking, followed by blocking in PBSBT (PBS, 5% BSA, 0.1% Triton X-100) at 37°C for 5 h. Primary antibodies diluted in PBSBT were applied and spheroids were incubated overnight at 4 °C (anti-FAP, CST #66562, 1:250; anti-γH2AX (phospho S139), Abcam ab26350, 1:250). Samples were washed five times in PBST (PBS, 0.5% Triton X-100; two quick washes, two washes for 5 min, and a final wash for 10 min) with gentle shaking and incubated with secondary antibodies (both 1:500 in PBSBT) for 3 h at room temperature with gentle shaking (donkey anti-rabbit Alexa Fluor 555, Abcam ab150074; goat anti-mouse Alexa Fluor 488, Abcam ab150113). After five PBST washes (as above), spheroids were incubated with ProLong™ Diamond Antifade Mountant with DAPI (P36962) overnight at room temperature with gentle shaking. Spheroids were then transferred to glass slides, covered with a coverslip, and sealed to prevent movement prior to imaging. Fluorescence images were acquired using a Leica SP8 confocal microscope equipped with a 40× objective. For each spheroid, a tiled scan was performed to cover the entire spheroid cross-section, and imaging was restricted to the central (mid-plane) optical section.

**SM. 8** **Conditioned-medium (CM) Experiment and Cytokine Analysis**

**SM. 8.1 Conditioned-medium (CM) Experiment**

CAF spheroids were generated by seeding CAFs at 4000 or 10000 cells/well. CAF spheroids were cultured for 48 h prior to treatment and then treated with [^161^Tb]Tb-FAPI-46 following the same spheroid treatment workflow described above. After 72 h of treatment, the CAF spheroid supernatant was collected and clarified by centrifugation to remove cells/debris, yielding CM. In parallel, PSN-1 spheroids (2000 cells/well) were generated and cultured for 48 h before CM transfer. The clarified CAF CM (for each CAF seeding density/ratio and treatment condition) was added to PSN-1 spheroids by complete medium replacement with equal volumes per well. PSN-1 spheroid growth was monitored up to day 13 (day 0 defined as the time of CM addition) by serial brightfield imaging.

**SM. 8.2 Cytokine profiling**

After brightfield imaging on day 13, culture supernatants were collected from each spheroid condition (three technical replicate wells per group) and clarified by centrifugation to remove cells/debris. 150 µL supernatant per condition was recovered and diluted with PBS to a final volume of 200 µL. Samples were shipped on dry ice to the UMC Utrecht MultiPlex Core Facility (MPCF) for cytokine (TGF-β1, IL-6 and VEGF) quantification using an in-house developed and validated (ISO9001) multiplex immunoassay based on Luminex xMAP technology (FLEXMAP 3D platform) [7]. Briefly, samples were incubated with antibody-conjugated MagPlex microspheres (1 h, room temperature, continuous shaking), followed by incubation with biotinylated detection antibodies (1 h) and phycoerythrin-conjugated streptavidin (10 min), with integrated wash steps between incubations. Data were acquired using xPONENT software and analysed by 5-parameter curve fitting using Bio-Plex Manager software.

**SM. 9 Quantitative Immunofluorescence Analysis** **of 3D Spheroids and Experimental Replication**

**SM. 9.1 Immunofluorescence image quantification of 3D spheroids**

Quantitative analysis of immunofluorescence signals was performed in QuPath (v0.5.0). Identical analysis settings were applied across all images within each experiment. Briefly, images were imported into QuPath, regions of interest (ROIs) were defined, nuclei were identified based on DAPI staining, and mean fluorescence intensity per cell was extracted for the relevant channels. Thresholding was kept constant across groups, and the resulting per-cell/per-ROI measurements were exported for downstream statistical analysis. For γH2AX foci analysis in 3D, γH2AX-positive objects were detected after nuclear segmentation. “Large” γH2AX foci were defined as γH2AX-positive objects with mean fluorescence intensity above a fixed threshold, determined empirically from representative images and then applied uniformly across all samples and conditions.

**SM. 9.2 Experimental Replication**

Experiments were performed with the number of independent repeats (biological replicates/independent experiments) as indicated below, and each condition included ≥3 technical replicate wells unless otherwise stated. For assays independently repeated multiple times with consistent trends, one representative experiment is shown for clarity. Specifically, CAF activation characterisation, cellular radioactivity experiments, 2D clonogenic assays, and 3D spheroid treatment/growth experiments were independently repeated three times (≥3 technical replicate wells per condition). 2D and 3D γH2AX immunofluorescence staining experiments were independently repeated two times (≥3 technical replicate wells per condition). Patient tissue staining, cytokine profiling, and the conditioned-medium experiment were performed once due to limited sample availability/material, and the number of samples/replicate wells used is specified in the corresponding figure legends.

**Supplementary Results (SR****)**

**SI Fig. 1 Extended examples of FAP immunofluorescence and [^161^Tb]Tb-FAPI-46 autoradiography in human PDAC tissues.** For each case, whole-section autoradiography images are displayed, with regions of relatively high uptake (red boxes) and low uptake (white boxes) indicated. Corresponding regions are shown at higher magnification as immunofluorescence images stained for α-smooth muscle actin (α-SMA, green), FAP (red), and nuclei (DAPI, blue).

**SI Fig. 2 FAP expression in CAFs, NF and PSN-1 and correlation with [^161^Tb]Tb-FAPI-46 uptake.** (A) Representative immunofluorescence images showing FAP staining (green) and nuclei (DAPI, blue) in CAFs, normal fibroblasts (NF), and PSN-1 cells. (B) Cellular uptake of [^161^Tb]Tb-FAPI-46 in CAFs, NF, and PSN-1, showing significantly higher uptake in CAFs compared with NF and PSN-1. (C) Quantification of FAP expression levels in CAFs, NF, and PSN-1 cells, indicating that the higher FAP expression in CAFs than in NF and PSN-1. CAFs = cancer-associated fibroblasts; NF = normal fibroblasts; FAP = fibroblast activation protein.

| **SI Table 1 Single-cell dosimetry of nuclear absorbed dose in CAFs and PSN-1 treated with [^161^Tb]Tb-FAPI-46 (10 vs 4000 kBq)** | | | | | | | | | | | | |
| --- | --- | --- | --- | --- | --- | --- | --- | --- | --- | --- | --- | --- |
| Cell type | Diameter (μm) | |  | AUC (Bq·s/cell) | | |  | S value to nucleus (mGy/Bq/s) | |  | Absorbed dose to nucleus (mGy) | |
|  | Cell | Nucleus |  | 10 | | 4000 |  | 10 | 4000 |  | 10 | 4000 |
| CAFs | 25 | 12 |  | 8.5 | | 401.5 |  | 0.2 | 0.1 |  | 1.7 | 40 |
| PSN-1 | 13 | 9 | |  | 3.4 | 184.8 |  | 2.1 | 2.1 |  | 7.1 | 388 |
| Average cell and nuclear diameters were used as geometric inputs for single-cell dosimetry. AUC_0–240 min_ was calculated from the uptake–time curves and used to derive the cumulated activity per cell. The absorbed dose to the nucleus was estimated using the nucleus as the target and reported as dose per unit activity and total nuclear absorbed dose for each activity condition | | | | | | | | | | | | |

**SI Fig. 3 Overall uptake/efflux kinetics and specificity of [^161^Tb]Tb-FAPI-46 at low vs high added radioactivity.** (A–B) Uptake and efflux pattern of [^161^Tb]Tb-FAPI-46 in CAFs and PSN-1 at (A) 0.5 nM (10 kBq) and (B) 200 nM (4000 kBq). (C–D) Specificity assessment under binding vs blocking conditions, separated into surface-bound and internalised fractions at (C) 0.5 nM (10 kBq) and (D) 200 nM (4000 kBq). In PSN-1 cells, uptake was dominated by surface binding at both conditions and was largely non-specific. In CAFs, the internalised fraction was predominantly specific at 0.5 nM, whereas at 200 nM uptake was dominated by surface binding and was largely non-specific. (E–F) Comparison of surface-bound and internalised [¹⁶¹Tb]Tb-FAPI-46 uptake under attached versus suspended cell conditions in (E) PSN-1 cells and (F) CAFs.

**SI Fig. 4 Longitudinal growth of 3D PSN-1/CAF spheroids following [^161^Tb]Tb-FAPI-46 treatment.** (A1–A5) Growth curves (growth ratio normalised to day 0) of spheroids composed of PSN-1 cells alone (A1), PSN-1/CAFs cocultures arranged as layer models at PSN-1:CAF ratios of 1:2 (A2) or 1:5 (A3), and mixed models at ratios of 1:2 (A4) or 1:5 (A5). Spheroids were exposed to 0, 500, 2000, or 4000 kBq [^161^Tb]Tb-FAPI-46 and monitored over 13 days. (B1–B5) The same datasets are displayed as grouped bar plots at each time point with multiple-comparison statistics versus the control group (0 kBq).

**References:**

1.	Nurmik M, Ullmann P, Rodriguez F, Haan S, Letellier E. In search of definitions: Cancer-associated fibroblasts and their markers. Int J Cancer. 2020;146:895-905. doi:10.1002/ijc.32193.

2.	Soon PSH, Kim E, Pon CK, Gill AJ, Moore K, Spillane AJ, et al. Breast cancer-associated fibroblasts induce epithelial-to-mesenchymal transition in breast cancer cells. Endocrine-Related Cancer. 2013;20:1-12. doi:10.1530/erc-12-0227.

3.	Wood R, Durali P, Wall I. Impact of Dual Cell Co-culture and Cell-conditioned Media on Yield and Function of a Human Olfactory Cell Line for Regenerative Medicine. Bioengineering (Basel). 2020;7. doi:10.3390/bioengineering7020037.

4.	Lindner T, Loktev A, Altmann A, Giesel F, Kratochwil C, Debus J, et al. Development of Quinoline-Based Theranostic Ligands for the Targeting of Fibroblast Activation Protein. Journal of Nuclear Medicine. 2018;59:1415-22. doi:10.2967/jnumed.118.210443.

5.	Huang Y, Zhang L, Wang M, Li C, Zheng W, Chen H, et al. Optimization of Precursor Synthesis Conditions of (2S,4S)4–[18F]FPArg and Its Application in Glioma Imaging. Pharmaceuticals. 2022;15:946.

6.	Chan CY, Chen Z, Guibbal F, Dias G, Destro G, O'Neill E, et al. [(123)I]CC1: A PARP-Targeting, Auger Electron-Emitting Radiopharmaceutical for Radionuclide Therapy of Cancer. J Nucl Med. 2023;64:1965-71. doi:10.2967/jnumed.123.265429.

7.	Mader N, Haghghi S, Klimek K, Schoeler C, Sarvestani MK, Rödel C, et al. Intensified therapy with 177Lu‐PSMA‐I&T in patients with high‐volume metastatic castration‐resistant prostate cancer. Journal of Nuclear Medicine. 2024;65:241176.
